# Comparison of microbiomes in ulcerative and normal mucosa of recurrent aphthous stomatitis (RAS)-affected patients

**DOI:** 10.1186/s12903-020-01115-5

**Published:** 2020-04-29

**Authors:** Zhongjun Yang, Qingyu Cui, Ran An, Juan Wang, Xiaobo Song, Yu Shen, Mingyu Wang, Hai Xu

**Affiliations:** 1 State Key Laboratory of Microbial Technology, Qilu Hospital, Shandong University, Qingdao, Shandong China; 2 College of Food Science and Pharmaceutical Engineering, Zaozhuang University, Zaozhuang, Shandong China; 3 Department of Medical Biology, Faculty of Health Sciences, University of Tromsø, Tromsø, Norway

**Keywords:** Recurrent apththous stomatitis, Microbiome, *Escherichia coli*, High throughput sequencing, Etiology

## Abstract

**Background:**

Recurrent aphthous stomatitis (RAS) is the most common form of oral ulcerative disease, whose cause is still unknown. Researchers have found the association of many factors with the occurrence of RAS, and proposed oral bacterial infection could be a cause for this disease.

**Methods:**

To investigate whether the occurrence of RAS is associated with oral bacterial infection, we performed high throughput sequencing analysis of bacterial samples collected from the normal oral mucosa and aphthous ulcers of 24 patients.

**Results:**

Firmicutes, Proteobacteria and Bacteriodetes were the most abundant phyla in the microbiomes analysed. The alpha diversities of the oral mucosa and aphthous ulcer microbiomes were similar, suggesting a similar richness and diversity. The NMDS analysis showed the oral mucosa and aphthous ulcer microbiomes are significantly different. This suggestion is further supported by Anosim, MRPP, and Adonis analyses. More detailed comparison of the two groups of microbiomes suggested that the occurrence of RAS is significantly associated with the increase of *Escherichia coli* and *Alloprevotella*, as well as the decrease of *Streptococcus*.

**Conclusions:**

Considering *E. coli* is a very common intestinal bacterium, we propose that *E. coli* colonization could be a cause for RAS, and controlling *E. coli* colonization could help curing RAS.

## Background

Recurrent aphthous stomatitis (RAS) is the most common form of oral ulcerative disease that affects as much as 5–20% of the population. It is characterized by shallow round ulcers that afflicts pain on the patients [1]. These lesions are benign and self-limiting, but are usually chronic and frequently recur [2], leading to difficulties in oral functions [3].

The etiology of RAS is still unclear, although association between RAS and a number of factors have been reported. These factors include local trauma [4], saliva composition changes [5], a series of systematic diseases such as HIV infection [6] and Crohn’s disease [7], genetic factors [8], food allergy [9], immunological factors [10], stress [11], nutritional deficiency [12], and microbial agents [13]. The lack of clear understanding of the etiology of RAS hinders the efficient treatment of this disease.

The role of several bacterial species in RAS has been implicated in previous investigations by culture-dependent techniques. *Helicobacter pylori* has been found on RAS lesions [14], and association between *H. pylori* and RAS has been suggested [13], although this association has been controversial [15]. Several *Streptococcus* species have been suspected to be involved in the development of RAS [16], and this involvement was suggested to be the result of autoimmune reaction of streptococcal heat-shock proteins [17]. Despite these investigations, no definitive connection between microbial infection and RAS has been demonstrated.

The emergence of high-throughput sequencing and various other high-throughput microbial techniques allowed in-depth culture-independent analysis of microbial colonization, and has been proven successful in detecting key pathogens for various diseases [18]. To date, several studies have been performed in attempt to understand the bacterial community composition in RAS-affected patients. Marchini et al. compared the microbiomes of 10 healthy and 10 RAS-affected subjects using 16S rDNA library-dependent cloning techniques, and found different microbiome structures [19]. Bankvall et al. compared the microbiomes of 60 healthy and 60 RAS-affected patients using Terminal-Restriction Fragment Length Polymorphism (T-RFLP) of 16S rDNA amplicons, and found differences in T-RFLP patterns, but were unable to pinpoint the key pathogens involved in RAS [20]. Seoudi et al. compared the saliva microbiomes of 26 healthy subjects and 8 RAS-affected patients using human oral microbe identification microarray analysis, and found decreased levels of *Rothia*, *Neisseria*, and *Veillonella* in RAS-affected patients [21]. Kim et al. compared the microbiomes of oral mucosa (*n* = 18) and saliva (*n* = 7) of RAS-affected patients with healthy subjects (n = 18) using 454 pyrosequencing of 16S rDNA, and found the association of the decrease of *Streptococcus salivarius* and the increase of *Acinetobacter johnsonii* with RAS risk [22]. Hijazi et al. performed 454 pyrosequencing of 16S rDNA from 18 RAS-affected patients and 17 healthy subjects, and found higher levels of Bacteroidales, Porphyromonadaceae and Veillonellaceae, along with decreased Streptococcaceae in association with RAS [23]. These investigations have a small sample size and cannot identify a significant difference (clustering) in the overall bacterial community between healthy and disease-affected samples, and have a lower sequencing depth (read numbers) and therefore could lead to missing information. Therefore, a more detailed comparison of microbiota between healthy and RAS-affected subjects is warranted.

In this work, we performed an in-depth analysis and comparison of the microbiomes between healthy mucosa and RAS lesions from 24 RAS-affected patients by high throughput Illumina sequencing of 16S rDNA, with an average sequence depth of 68,633 reads per sample. Suggestions on the association of specific bacteria with RAS are made that require further mechanistic investigations for the confirmation of etiology.

## Methods

### Sample collection and DNA extraction

Bacterial samples were taken from the surface of normal oral mucosa and aphthous ulcers from 24 RAS patients in the Second Hospital of Shandong University and Stomatological Hospital of Shandong University using sterile cotton in 2018. RAS patients that did not take antibiotics at least 3 months prior to the sampling were recruited as participants of the experiments (Additional file 1). Systematic diseases and other medication were not considered. Approximately 50% of the participants were male (male: female = 13: 11). Most of the participants are mid-aged or older (20–30: 3/24, 30–40: 7/24, 40–50: 6/24; above 50: 8/24). RAS was diagnosed by the authors following criteria previously documented in literature [1]. Most of the lesions are single episodes (19/24, one ulcer per person), and are minor RAS (20/24, remainders being major RAS). The number of patients was arrived at based on requirement of the high-throughput microbiome sequencing technique that generally requires at least 20 samples for clear conclusions. The site of sample collection for healthy mucosa is on the opposite side of the ulcers to avoid cross-contamination. The heads of the cotton swabs were cut off with sterile scissors, soaked in 0.9% NaCl, and vortexed for 5 min. The total DNA was subsequently extracted with Plant Genomic DNA Kit (Tiangen Biotech (Beijing) Co., Ltd., Beijing China). The DNA content was determined prior to high throughput sequencing.

### 16S rDNA high throughput sequencing and data analysis

The V4-V5 region of 16S rDNA was PCR amplified from extracted DNA samples for the determination of bacterial community composition by high throughput sequencing using Illumina HiSeq2500 PE250 (Illumina Inc., San Diego, CA). Raw tags were obtained using FLASH V1.2.7, and processed using Qiime V1.9.1 to obtain clean tags. Chimera were removed using the UCHIME algorithm and Gold database to obtain effective tags. This was performed to obtain effective tags and to exclude potential bias introduced during read generation. The effective tags were grouped into Operational Taxonomic Units (OTUs) with a 97% sequence identity cutoff using Uparse V7.1.1001. Annotation of the taxonomy of each OTU was performed using the Mothur method and the SILVA database. The levels of each OTU were normalized for further analysis of alpha and beta diversity.

The alpha diversity indexes were calculated using Qiime V1.9.1. Rarefaction curves were drawn using R V2.15.3. Weighted Unifrac distances were calculated using Qiime V1.9.1. Anosim, MRPP and Adonis analyses were performed using the vegan package of R software. NDMS analysis was performed using the vegan package of the R software. LEfSe analysis was performed using the LEfSe software with a default LDA score cutoff of 4.

### Statistics

Two-tailed *t*-tests were performed for the comparison of bacterial community composition between oral mucosa microbiomes and aphthous ulcer microbiomes.

### Ethics

The experiments in this study were conducted in accordance with the Declaration of Helsinki, and were approved by the Scientific Ethics Committee of Qilu Hospital, Shandong University. Consent to participate was obtained from all subjects verbally as samples were taken from outpatients in scenarios where patients’ time is limited for the purpose of preparing necessary written documents. This procedure was approved by the Scientific Ethics Committee of Qilu Hospital.

## Results

### The bacterial community compositions of oral mucosa and aphthous ulcers

In order to understand the bacterial community compositions of oral bacterial and aphthous ulcers, we collected bacterial samples from normal oral mucosa and aphthous ulcers from 24 patients using cotton swabs. Total DNA was extracted from these samples, from which 16S rDNA was amplified and sequenced by high throughput sequencing. An average sequence depth of 68,633 reads per sample was obtained. These reads were grouped into Operational Taxonomic Units (OTUs) with 97% sequence identity as cutoff. An average of 570 OTUs/sample were found.

**Fig. 1:**
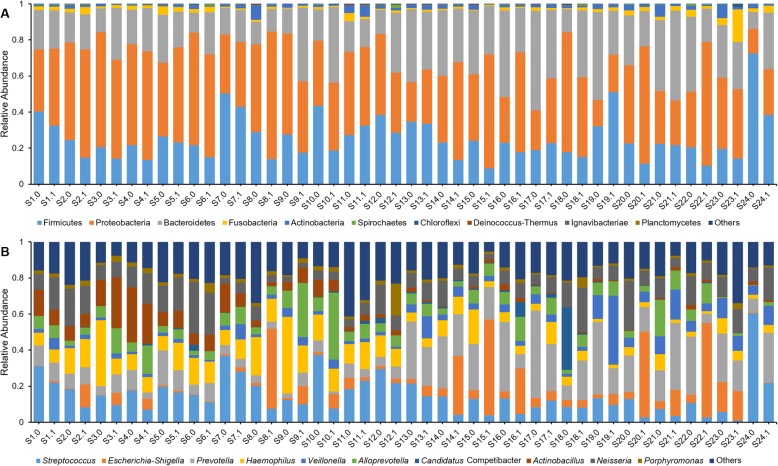
The bacterial community composition of oral mucosa and aphthous ulcers. **a** bacterial community composition on the phylum level; **b** bacterial community composition on the genus level. Sx.0 indicates the oral mucosa of subject x; Sx.1 indicates the aphthous ulcers of subject x

The majority of oral bacteria found in this study belong to three phyla: Firmicutes, Proteobacteria and Bacteroidetes (Fig. 1a). The 10 most abundant genera are *Streptococcus*, *Prevotella*, *Haemophilus*, *Neisseria*, *Actinobacillus*, *Alloprevotella*, *Veillonella*, *Escherichia-Shigella*, *Candidatus* Competibacter, and *Porphyromonas* (Fig. 1b). It is worth noting that the top 3 phyla represent 99.41% of all bacteria, and the top 10 genera represent 81.90% of all bacteria.

### Alpha diversity of oral and aphthous ulcerative microbiomes

**Fig. 2:**
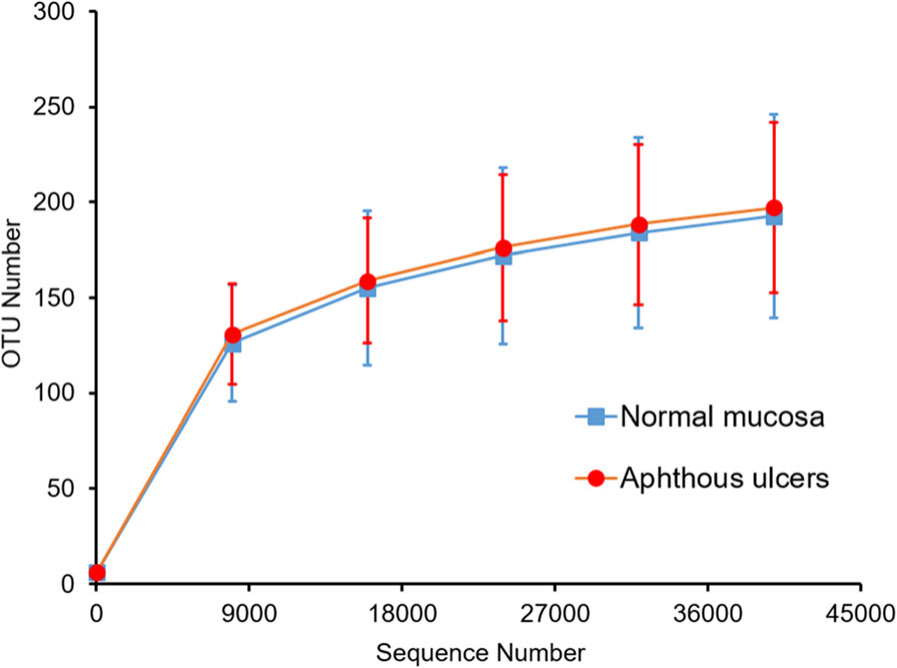
The Rarefaction curve of normal mucosa and aphthous ulcer microbiomes. Error bar indicates standard deviation (*n* = 24)

The alpha diversity indexes that indicate the community richness and diversity of bacteria in oral mucosa and aphthous ulcers were calculated. The observed species index explains the number of species identified in a bacterial community. Shannon and Simpson indexes are indicators for the diversities of bacterial communities, while the Chao1 and Abundance-based Coverage Estimation (ACE) estimators are indicators for the richness of bacterial communities. The rarefaction curves of the two groups of microbiomes suggest similar richness and diversity (Fig. 2). This observation is echoed by the finding that the Shannon indexes, Simpson indexes, Chao1 estimators, ACE estimators were similar between the two groups of microbiomes (Table 1). More detailed comparison of these indexes between oral and aphthous ulcerative microbiomes of the same individual further confirmed this finding: Although differences can be found between each pair of compared microbiomes for several subjects (such as Shannon index for individual S15 and Chao1 estimator for individual S10), the indexes are close for the same subject for the majority of subjects investigated. These findings suggest that the alpha diversity of microbiomes in oral mucosa and aphthous ulcers are similar.

**Table 1:** Alpha diversity indexes

Experimental group	Observed species	Shannon index	Simpson index	Chao1 estimator	ACE estimator
Normal mucosa	198	3.68	0.836	219.404	222.88
Aphthous ulcers	203	3.681	0.843	234.721	234.363

### Analysis of the bacterial community compositions of oral mucosa and aphthous ulcers

**Fig. 3:**
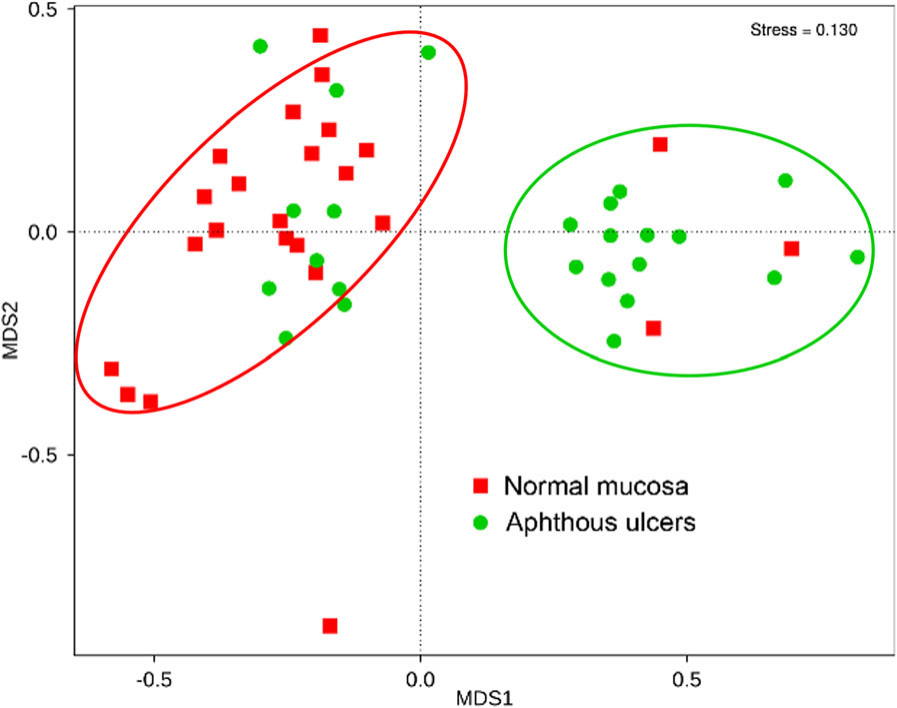
NMDS analysis of investigated bacterial communities. The red oval covers the majority of normal mucosa samples. The green oval covers the majority of aphthous ulcers

The Non-Metric Multi-Dimensional Scaling (NMDS) analysis was performed on the bacterial community compositions of oral mucosa and aphthous ulcers (Fig. 3). The oral mucosa and aphthous ulcer groups are clearly distinguished, suggesting a significant difference between the bacterial community compositions of the two types of microbiota. This is further confirmed by Anosim (*p* = 0.009), MRPP (*p* = 0.004), and Adonis (*p* = 0.004) analyses, all suggesting the differences between the oral mucosa and aphthous ulcer groups are significantly bigger than within each group. These results suggest a clear and significant difference between the bacterial community compositions of oral mucosa and aphthous ulcers, although the richness and diversity indexes of their bacterial communities are largely similar.

### Significantly different bacterial groups between the microbiomes of normal oral mucosa and aphthous ulcers

**Fig. 4:**
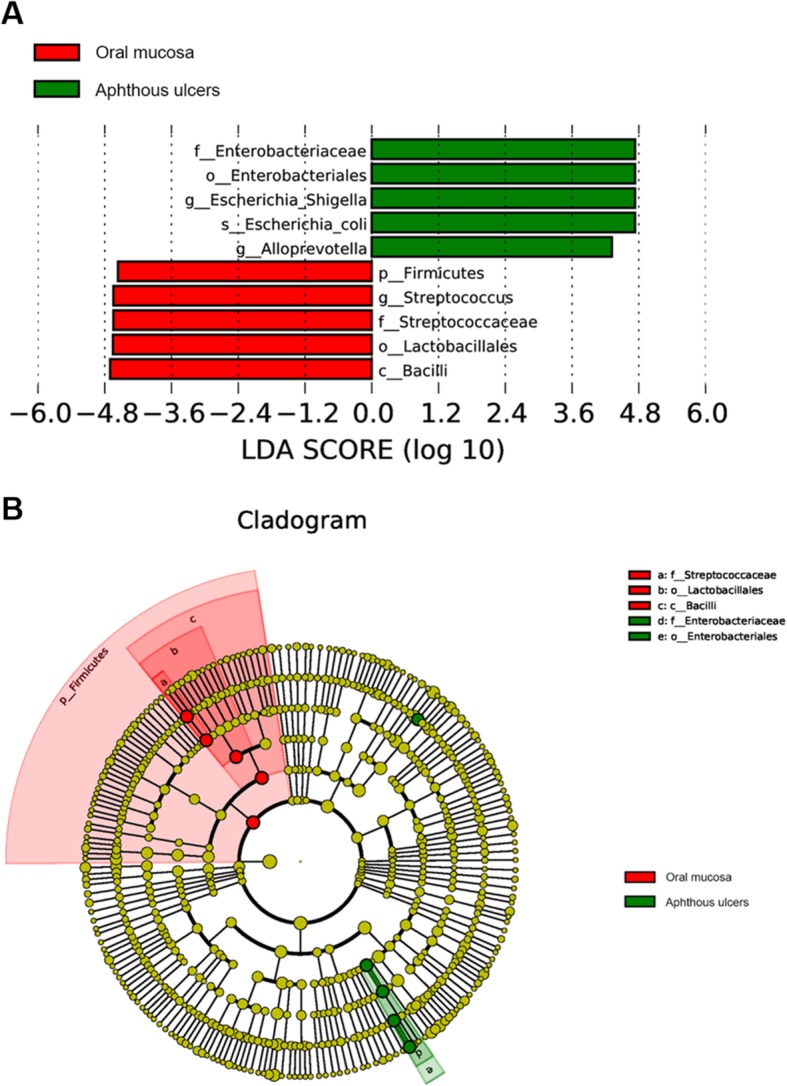
LEfSe analysis of investigated bacterial communities. **a** the LDA scores of significant biomarkers; **b** The Cladogram of significant biomarkers. The diameters of each circle are proportional to its relative abundance

The LEfSe (LDA Effect Size) analysis searches for statistically significant difference in metagenomics. This analysis was performed between the oral mucosa microbiomes and the aphthous ulcer microbiomes (Fig. 4). Significantly more represented in normal mucosa samples are Firmicutes on the phylum level, Bacilli on the class level, Lactobacillales on the order level, Streptococcaceae on the family level, and *Streptococcus* on the genus level (Fig. 4a). Significantly more represented in aphthous ulcer samples are Enterobacteriales on the order level, Enterobacteriaceae on the family level, *Escherichia-Shigella* and *Alloprevotella* on the genus level, and *Escherichia coli* on the species level (Fig. 4b). From the Cladogram analysis, it can be seen that *Streptococcus* is significantly enriched of the oral mucosa microbiome, while *Streptococcus* and *Alloprevotella* are significantly enriched in the aphthous ulcer microbiome (Fig. 4b).

Statistical comparison between taxonomic abundances in oral mucosa and aphthous ulcer microbiomes confirmed the roles of *Streptococcus* (*p* = 0.0077, mucosa group 14.27–24.11% and aphthous ulcer group 7.88–13.93% at 95% Cl), *E. coli* (*p* = 0.0031, mucosa group 1.60–4.40% and aphthous ulcer group 7.93–21.99% at 95% Cl) and *Alloprevotella* (*p* = 0.0427, mucosa group 4.05–7.17% and aphthous ulcer group 6.24–12.89% at 95% Cl) (Additional file 2). Other significantly enriched taxa among the ten most abundant taxa at each taxonomic level include: 1) Enriched in oral mucosa microbiomes: Actinobacteria (*p* = 0.014, mucosa group 1.71–3.35% and aphthous ulcer group 0.84–1.75% at 95% Cl) at class level and Bacillales (*p* = 0.042, mucosa group 1.72–3.37% and aphthous ulcer group 1.06–2.00% at 95% Cl) at order level; 2) Enriched in aphthous ulcer microbiomes: Porphyromonadaceae (*p* = 0.046, mucosa group 1.35–1.74% and aphthous ulcer group 1.66–4.35% at 95% Cl) at family level and *Porphyromonas* (*p* = 0.029, mucosa group 1.28–1.66% and aphthous ulcer group 1.56–4.25% at 95% Cl) at genus level.

## Discussion

Understanding the etiology of RAS is a big step forward in finding effective cures for this common disease, and it has been suspected that microbial infection contributes to RAS [13, 16]. Recent progress in high throughput sequencing techniques enables metagenomic approaches in understanding the microbiomes of biological samples, therefore allows us to pinpoint the specific pathogen responsible for diseases by comparing the microbiomes of pathological and normal tissues. Therefore, we exploited high-throughput sequencing technologies in this work in attempt to find specific association of bacterial community compositions with aphthous ulcers, which further leads to proposals of the etiology of RAS.

In this work, we found that the increase of *E. coli* and *Alloprevotella*, as well as the decrease of *Streptococcus* in bacterial communities is significantly associated with aphthous ulcers. The decrease of *Streptococcus* in aphthous ulcers is in agreement with previous findings [22, 23]. However, the increase of *E. coli* in aphthous ulcers is a new and particularly intriguing finding. *E. coli* is one of the most common bacteria in the human microbiome, particularly intestinal microbiome [24]. Inoculation of *E. coli* to oral mucosa is easy and common via the fecal-oral pathway. Considering 40% of the human population suffers from RAS, the cause of this disease has to be a common factor. This common occurrence is in coincidence with *E. coli* colonization which is also a very common phenomenon. Therefore, the significant association of aphthous ulcers with *E. coli* abundance leads to the proposal that *E. coli* colonization could be the cause of RAS. Previous investigations suggested that *Helicobacter pylori* could be the cause of RAS, but results from this work do not suggest a significant correlation between aphthous ulcers and *H. pylori* (*p* = 0.185). Therefore, we doubt that *H. pylori* has a direct role in the formation of aphthous ulcers, in agreement with the previous suggestion that *H. pylori* does not play a role in RAS [15].

A number of previous studies investigated the microbiota of RAS [18–23]. These investigations are mostly qualitative rather than quantitative, and cannot lead to the identification of significantly enriched groups in the bacterial community of aphthous ulcers. Three previous studies quantified the microbial abundance of bacteria in aphthous ulcers using microarray or pyrosequencing approaches [21–23]. These investigations either compared the oral bacterial community composition of healthy subject and patients and therefore suffered from background noise caused by differences between individuals [22], involved saliva microbiome which could naturally have different bacterial community composition with the mucosa as saliva is a natural disinfectant [21, 22], or has a relatively small sample size (*n* = 8 or 12) [21, 23]. In particular, the two investigations with pyrosequencing only had respectively 3000 and 9500 tags/sample [22, 23], which could lead to loss of information due to lower sequencing depth and smaller sample volume. The methods used in this work ruled out differences between individuals by comparing the normal oral mucosa and aphthous ulcers of the same individual, had a larger sample volume (*n* = 24), and had a better sequencing depth (> 68, 000 tags/sample). Therefore, we are able to more effectively detect bacterial groups specific to aphthous ulcers in this work. It needs to be noted that samples were taken from only RAS patients intentionally without collecting samples from healthy individuals as controls, because it was decided that individual diversity may contribute significantly towards differences in bacterial community leading to difficulties in finding bacteria that are associated with RAS. Including healthy individuals will only complicate the study rather than help it. Also, not having healthy controls does not weaken the findings of this work as this work aims to find RAS-associated, localized, ulcerative mucosa-bearing microbes, and a proper control is the healthy mucosa of the same individual. A large number of taxa were found differently represented in oral mucosa and aphthous ulcers (Additional file 2). With more stringent statistical analysis like LEfSe, we are capable of identifying *E. coli* and *Alloprevotella* as the bacterial groups specific to aphthous ulcers, which was never observed before. We also confirmed previous findings that the reduction of *Streptococcus* (Streptococcaceae) and *Rothia* is associated with aphthous ulcers [22, 23], while the increase of Porphyromonadaceae is associated with aphthous ulcers [23]. Previous reports on the positive association of *Acinetobacter* and Bacteroidales with aphthous ulcers, as well as the negative association of *Neisseria* with aphthous ulcers were not confirmed by our results [21–23]. The role of Veillonellaceace on aphthous ulcers was controversial [21, 23], and our results couldn’t suggest a significant correlation between this group of bacteria with RAS.

The work we performed suggested that the colonization of *E. coli* or *Alloprevotella*, more likely the former, may be the cause of RAS. However, it needs to be pointed out that this suggestion is not conclusive, as finding an association is not equivalent to finding the causality. We cannot rule out the possibility that RAS leads to increased abundance of *E. coli* and *Alloprevotella*, in contrary to our hypothesis that increased abundance of *E. coli* and/or *Alloprevotella* leads to RAS. Furthermore, consideration on other possible complications influencing oral environment and bacterial community structures, such as other underlying conditions and drug use, was not included in this investigation, which was due to the assumption that they are not major drivers of the oral microbiomes and these effects may be minimized by stringent statistics. A much larger surveillance is still needed to identify detailed factors influencing oral microbiomes. Nevertheless, the findings of this work, in particularly the coincidence that *E. coli* colonization and RAS occurence are both common, points to a high possibility to the etiology of RAS. Further in-depth pathological work is needed to confirm this possibility. These findings have the potentials to guide the discovery of new cures for RAS, which may include targeting oral *E. coli* colonization and removing it using antibiotics.

## Conclusions

In conclusion, we compared the microbiomes of normal oral mucosa and aphthous ulcers of 24 subjects by high throughput sequencing, and identified bacterial groups that represent both oral mucosa and aphthous ulcers. A novel proposal was made that *E. coli* or *Alloprevotella*, more likely the former, may be the cause of RAS. This work can provide a new road for finding the etiology of RAS, which will help searching for effective cures to this common disease. Limitations of this work still exist including the inability to find out whether the change of oral microbiomes is the reason or the result of RAS, and the limit on sample size which prevents us from carrying out analysis on more factors that potentially affect oral microbiome. Further larger surveillance and more in vivo experimentation is warranted to address these limitations in the future.

## Supplementary information

**Additional file 1: Table S1.** Characteristics of patients in this study.

**Additional file 2.** Significantly differentially represented taxa in oral mucosa microbiomes and aphthous ulcer microbiomes (by t-test).

## Data Availability

The datasets used and/or analysed during the current study are available from the corresponding author on reasonable request.

## Abbreviations

RAS: Recurrent aphthous stomatitis
T-RFLP: Terminal-Restriction Fragment Length Polymorphism
OTU: Operational Taxonomic Unit
NMDS: Non-Metric Multi-Dimensional Scaling
LEfSe: LDA Effect Size
