# Supplementary material for: Comparison of microbiomes in ulcerative and normal mucosa of recurrent aphthous stomatitis (RAS)-affected patients
Source: BMC Oral Health. 2020 Apr 29;20:128. doi: 10.1186/s12903-020-01115-5 (PMC7189554; doi:10.1186/s12903-020-01115-5)
Supplement: Supplementary file 1 — Additional file 1: Table S1. Characteristics of patients in this study. [file 12903_2020_1115_MOESM1_ESM.docx]

**Table S1 Characteristics of patients in this study.**

| **Age** | 20-30: 3 persons |
| --- | --- |
|  | 30-40: 7 persons |
|  | 40-50: 6 persons |
|  | 50 and above: 8 persons |
| **Gender** | Male: 13 persons |
|  | Female: 11 persons |
| **Type of RAS** | Minor: 20 persons |
|  | Major: 4 persons |
| **Time for RAS affliction** | <0.5 years: 11 persons |
|  | 0.5-1 years: 6 persons |
|  | >1 year: 7 persons |
| **Number of ulcers** | Single ulcer: 19 persons |
|  | Multiple ulcers: 5 persons |
